# Broad-spectrum molecular detection of zoonotic agents in wild micromammals: a nationwide surveillance study in the Republic of Korea

**DOI:** 10.1186/s13071-026-07585-w

**Published:** 2026-07-26

**Authors:** Hyunyoung Yoon, Hak Seon Lee, Ji Ye Seo, Jin Seo Park, Hee Il Lee, Seong Yoon Kim

**Affiliations:** 1 Division of Vectors and Parasitic Diseases, Korea Disease Control and Prevention Agency, 187 Osongsaengmyeong 2-ro, Osong-eup, Heungdeok-gu, Cheongju, Chungcheongbuk-do 28159 Republic of Korea; 2 Present Address: Infectious Disease Inspection Division, Animal and Plant Quarantine Agency, 46 Deungchon-ro 39ga-gil, Gangseo-gu, Seoul, 07670 Republic of Korea; 3 Present Address: Department of Veterinary Medicine and Public Health, Armed Forces Medical Research Institute, 90bun, Jaun-ro, Yuseong-gu, Daejeon, 34059 Republic of Korea

**Keywords:** Micromammal-borne zoonotic pathogens, TaqMan Array Card, Zoonoses, Molecular surveillance, Republic of Korea

## Abstract

**Background:**

Wild micromammals serve as key hosts for diverse zoonotic pathogens, maintaining these agents in natural ecosystems and facilitating potential spillover to humans. Assessing pathogen prevalence in micromammals is essential for evaluating zoonotic risk and informing public health interventions.

**Methods:**

In this study, we applied the TaqMan Array Card (TAC) platform, a high-throughput molecular diagnostic tool, to micromammal-borne zoonotic pathogen surveillance. From October to November 2022, a total of 160 wild micromammals, including *Apodemus agrarius* (Striped field mouse) (90.6, *n* = 145) and *Crocidura lasiura* (Ussuri white-toothed shrew) (9.4%, *n* = 15), were collected from 12 regions. Spleen tissues were screened for 45 viral, bacterial, and protozoan pathogens using the TAC platform.

**Results:**

Viral pathogens were detected at lower frequencies, including Junin virus (2.5%, *n* = 4) and lymphocytic choriomeningitis virus (0.6%, *n* = 1). In contrast, bacterial pathogens showed high prevalence, including *Bartonella* spp. (78.8%, *n* = 126), *Ehrlichia* spp. (47.5%, *n* = 76), *Anaplasma* spp. (46.9%, *n* = 75), *Borrelia* spp. (36.3%, *n* = 58), *Orientia tsutsugamushi* (25.6%, *n* = 41), *Rickettsia* spp. (4.4%, *n* = 7), and *Leptospira* spp. (2.5%, *n* = 4) as well as protozoan pathogens such as *Trypanosoma* spp. (87.5%, *n* = 140), *Cryptosporidium* (20.0%, *n* = 32), and *Toxoplasma gondii* (1.3%, *n* = 2). However, some detections particularly viral pathogens were based solely on the TAC platform and were not confirmed by conventional PCR and sequencing. Phylogenetic analyses of TAC-positive samples revealed the presence of diverse zoonotic agents, including *Anaplasma phagocytophilum*, *Ehrlichia muris*, *Candidatus* Ehrlichia khabarensis, *Candidatus* Neoehrlichia mikurensis, *Borreliella afzelii*, *Borrelia miyamotoi*, *O. tsutsugamushi* Saitama and Young-worl strains, *Bartonella quintana*, *B. fuyuanensis*, and *B. japonica*.

**Conclusions:**

These findings demonstrate the widespread regional distribution of micromammal-borne zoonotic pathogens across the ROK and provide essential baseline data to support integrated One Health surveillance frameworks for emerging and re-emerging micromammal-borne zoonoses.

**Graphical Abstract:**

**Figure:**
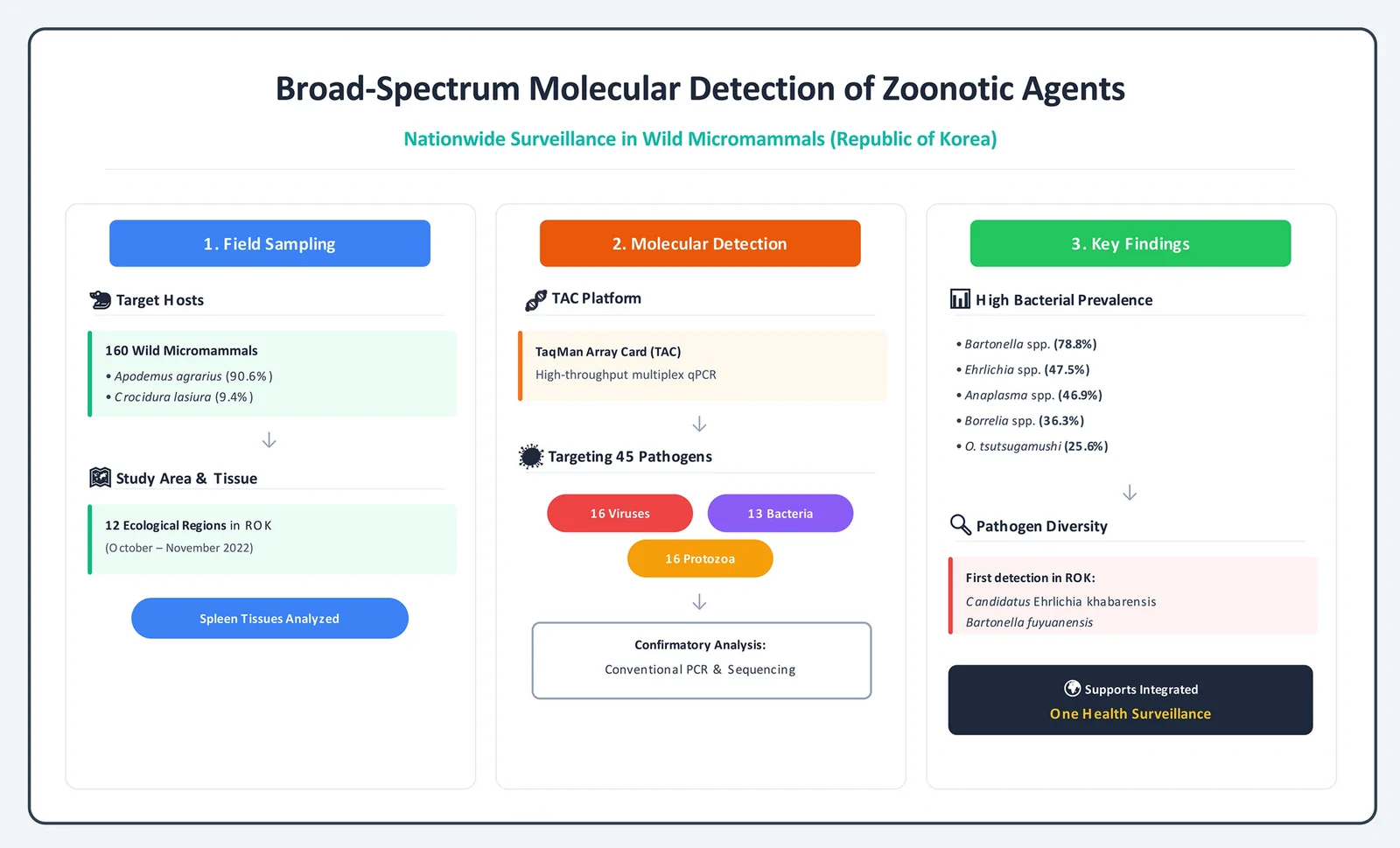

**Supplementary Information:**

The online version contains supplementary material available at https://doi.org/10.1186/s13071-026-07585-w.

## Background

Environmental and societal changes, including climate change, globalized trade, urbanization, and increased human mobility, are rapidly eroding traditional geographic barriers to pathogen transmission [1]. Consequently, pathogens that cause disease are spreading in new regions. For example, Japan, historically free of dengue virus transmission, recently reported a dengue fever outbreak [2]. Emerging infectious diseases have become one of the most critical public health challenges of the twenty-first century, posing significant risks not only to human health but also to ecosystem stability [3]. The increasing frequency and global spread of zoonotic disease outbreaks underscore the need for proactive surveillance and early detection systems.

Among wildlife species involved in zoonotic disease transmission, micromammals are of particular concern because of their high reproductive rates, broad ecological distribution, frequent cohabitation with humans, and well-established roles as reservoirs and vectors for diverse zoonotic pathogens [4]. In addition, climate and land-use changes alter micromammal population dynamics and geographic distribution, thereby influencing the spatiotemporal patterns of pathogen transmission [4, 5]. These pathogens include viruses such as hantavirus and Lassa virus, bacteria such as *Bartonella* spp. and *Leptospira* spp., and protozoan parasites such as *Trypanosoma* species [6]. Given the central role micromammals play in zoonotic disease ecology, effective detection and monitoring of the diverse pathogens from micromammals is essential.

Several micromammal-associated zoonotic diseases, including hemorrhagic fever with renal syndrome (HFRS), leptospirosis, spotted fever group rickettsiosis, and epidemic typhus, are reported annually in the Republic of Korea (ROK). Between 2001 and 2017, the average incidence of HFRS was approximately 0.8 cases per 100,000 population, with a mean case fatality rate of 1.3% [7]. A study targeting 156 wild micromammals across 27 regions reported substantial pathogen prevalence, including *Bartonella* spp. (46.8%), *Coxiella burnetii* (16.0%), *Leptospira interrogans* (15.4%), *Anaplasma phagocytophilum* (13.5%), and Severe fever with thrombocytopenia syndrome virus (SFTSV) (5.8%) [8]. In urban settings, surveillance documented the circulation of micromammal-borne viruses by capturing nearly 1,950 *Rattus norvegicus* in Seoul between 2006 and 2010 and serologically screening them for Seoul virus, with seropositivity detected in approximately 4.7% of tested micromammals [9]. More recently, national-scale surveillance efforts carried out in 2022–2023 have identified *A. phagocytophilum* in 10.3%, *Ehrlichia* spp. in 0.2–0.5%, and *Rickettsia raoultii* in 2.9% of micromammals [10]. Despite these efforts, prior investigations have often been limited by restricted geographic coverage or a narrow pathogen focus, rarely encompassing viruses, bacteria, and protozoa simultaneously across broad spatial scales. This constrained scope limits understanding of the full diversity of micromammal-borne zoonotic threats, including pathogens with potential for emergence or introduction through international travel and trade.

Among available molecular diagnostic platforms, the TaqMan Array Card (TAC) enables high-throughput multiplex pathogen detection. TAC is a microfluidic real-time PCR system that simultaneously and rapidly amplifies nucleic acids from multiple pathogens using preloaded pathogen-specific primers and probes, enabling specific and sensitive detection of target pathogens from a single specimen [11, 12]. Previous studies have demonstrated that TAC provides analytical performance comparable to reference real-time PCR assays and, in some cases, higher sensitivity than commercial multiplex pathogen detection platforms [13, 14]. Furthermore, TAC achieved an overall diagnostic accuracy of 93.0%, with 96.4% sensitivity and 87.7% specificity compared with standard PCR methods [12]. In addition, unlike multiplex PCR, in which primer competition may occur within a single reaction, TAC employs separate reaction wells that minimize cross-reactivity and allow independent optimization of primer–probe sets, which may contribute to improved reproducibility and assay consistency [15]. DNA microarray platforms, another high-throughput molecular approach, have also been applied to pathogen screening in micromammals [16]; however, while microarrays enable broad-spectrum detection, TAC is optimized for precise multiplex detection of selected targets with high analytical accuracy [17, 18]. This technology has been successfully applied in public health and environmental surveillance, including investigations of gastrointestinal infections [19], central nervous system pathogens [20], respiratory pathogens [17], and wastewater-based epidemiology [21]. The capability of TAC makes it particularly suitable for wildlife surveillance, where co-infections and pathogen diversity are common. In the context of increasing risk of pathogen spillover from wildlife to humans, multi-pathogen screening tools are essential for strengthening One Health surveillance frameworks. Consistent with this need, a recent molecular study of Korean water deer revealed previously unrecognized pathogen diversity in natural wildlife, underscoring the importance of large-scale surveillance approaches [22].

To address these gaps, we aimed to systematically assess the prevalence and geographic distribution of viral, bacterial, and protozoan pathogens in wild micromammals across the ROK. We aimed to employ TAC to conduct a large-scale molecular survey of micromammal samples collected from 12 regions in 2022. The rationale was to establish a baseline for micromammal-borne zoonoses in the ROK and provide evidence to inform national preparedness and biosecurity strategies aimed to mitigate risks by both endemic and emerging pathogens.

## Methods

### Micromammal trapping and sample collection

All animal handling and experimental procedures were approved by the Institutional Animal Care and Use Committee (IACUC) of the Korea Disease Control and Prevention Agency (KDCA) (approval number: KDCA-IACUC-21-002). Following permission obtained under the Wildlife Protection and Management Act, wild micromammals were collected from 12 ecologically diverse regions across the ROK, including Goseong, Goheung, Donghae, Sejong, Yeoju, Uiseong, Jeongeup, Jeju, Seogwipo, Cheongdo, Pocheon, and Haenam during October and November 2022. Trapping was conducted using Sherman folding live traps (3 × 3.5 × 9 inches; BioQuip, CA, USA) baited with peanut butter-coated biscuits. Traps were positioned at 3–5-m intervals and retrieved the following day [23]. All captured micromammals were transported to the laboratory immediately after retrieval and euthanized within 6 h of capture. During this interval, each individual remained housed separately in the original trap until euthanasia. Euthanasia was performed in a dedicated chamber using carbon dioxide (CO₂) inhalation with a gradual fill method, introducing CO₂ at a displacement rate of 20.0–30.0% of the chamber volume per minute until a final concentration of approximately 70.0% was achieved and respiration had completely ceased [24]. Death was confirmed by the absence of heartbeat and pedal reflex prior to dissection [25]. Immediately after euthanasia, animals were placed in dorsal recumbency, and a midline abdominal incision was performed aseptically inside a biological safety cabinet. The entire spleen and both kidneys were aseptically excised using sterile instruments. Each tissue sample was transferred into sterile cryovials and stored at − 80 °C until subsequent molecular analyses.

### RNA extraction from tissues

Genomic RNA was extracted from micromammal tissues. Approximately 10 mg of tissue was placed in 400 μL of lysis buffer containing 2.8 mm ceramic (zirconium oxide) beads and homogenized using a Precellys Evolution tissue homogenizer (Bertin Technologies, Montigny-le-Bretonneux, France) for two cycles of 30 s at 7500 rpm. After homogenization, 40 μL of chloroform was added to each sample to remove RNase contaminants, according to the manufacturer’s instructions. Subsequently, 400 μL of isopropanol was added, and samples were gently mixed for 2 min to precipitate nucleic acids. Genomic RNA was then extracted using the MagMAX^™^ mirVANA^™^ Total RNA Isolation Kit (Applied Biosystems, Foster City, CA, USA) according to the manufacturer’s protocol, using the KingFisher™ automated extraction system (Thermo Fisher, Waltham, MA, USA).

### TaqMan array card (TAC) assay for multiple pathogen detection

A custom-designed TAC (Thermo Fisher, Waltham, MA, USA) was employed for the simultaneous detection of 45 micromammal-borne pathogens, comprising 16 viral species, 13 bacterial species, and 16 protozoan parasites selected based on zoonotic potential, prior detection in micromammals, and public health relevance (Supplementary Table S1). A total of 5 μL of RNA extracted from each specimen was subjected to reverse transcription and template-specific preamplification using a customized primer pool and TaqPath™ 1-Step Master Mix (Thermo Fisher, Waltham, MA, USA), performed over 14 PCR cycles to enhance assay sensitivity. The resulting cDNA was combined with TaqMan^™^ Fast Advanced Master Mix (Thermo Fisher, Waltham, MA, USA), and 100 μL of the reaction mixture was loaded into each sample port of the TAC card. Cards were centrifuged at 1200 rpm for 1 min, repeated twice, to ensure uniform distribution of samples across the microfluidic reaction wells. Cards were then sealed using a TAC sealer to prevent evaporation and maintain reaction integrity during thermal cycling, and the sample loading ports were trimmed to ensure optimal instrument performance [11].

Real-time quantitative PCR (qPCR) was performed using the QuantStudio^™^ Dx Real-Time PCR System (Thermo Fisher, Waltham, MA, USA) under the following conditions: initial activation at 95 °C for 10 min, followed by 40 cycles of 95 °C for 3 s and 60 °C for 30 s. Data acquisition and analysis were performed using Design & Analysis Software 2 (Thermo Fisher, Waltham, MA, USA). Amplification was considered valid when the fluorescence signal crossed the threshold line. Samples were classified as positive for the target pathogen when the Cq confidence value was ≥ 0.7 and the amplification score (Amp Score) was ≥ 1.0.

### Conventional PCR amplification

Conventional PCR was performed for confirmatory reanalysis to obtain sequences from TAC-positive samples, excluding protozoan pathogens. Reactions were conducted using AccuPower^®^ PCR Premix (BIONEER, Daejeon, ROK) and a ProFlex PCR System (Thermo Fisher, Waltham, MA, USA). For nested PCR, 1 μL of the primary PCR product was used as the template for secondary amplification. Amplified products corresponded to partial regions of the following target genes: the S segment (JUNV; Junin virus; LCMV; lymphocytic choriomeningitis virus), the L gene (JUNV), the 16S rRNA gene (*Bartonella*, *Ehrlichia*, *Anaplasma*), the *flaB* gene (*Borrelia*), the 56-kDa type-specific antigen (*tsa*) gene (*Orientia tsutsugamushi*), the *gltA* gene (*Rickettsia*), and the *rpoB*, *lipL32*, and *gyrB* genes (*Leptospira*). Primer sequences and amplicon sizes are provided in Supplementary Table S2 [69–82].

Additional molecular analyses were conducted to identify wild micromammal species. Genomic RNA was extracted from kidney tissues, and species identification was performed by amplifying a partial region of the gene encoding mitochondrial *Cytochrome b*, a molecular marker widely used in micromammal phylogenetic studies [26, 27]. PCR reactions were performed using the same AccuPower^®^ PCR Premix and ProFlex PCR System under the following thermal cycling conditions: initial denaturation at 94 °C for 5 min; 30 cycles of denaturation at 94 °C for 30 s, annealing at 48 °C for 30 s, and extension at 72 °C for 1 min; followed by a final extension at 72 °C for 5 min. The primers used were Cy-14724F (5′-GACYARTRRCATGAAAAAYCAYCGTTGT-3′) and Cy-15909R (5′-CYYCWTYIYTGGTTTACAAGACYAG-3′), which are widely used mammal-specific primers validated for selective amplification of this marker in micromammals.

### Sequencing and phylogenetic analysis

PCR products yielding positive results were purified and sequenced by a commercial service (SolGent, Daejeon, ROK). Raw chromatograms were assembled into consensus sequences using Chromas Software 2.33, and species identification was performed by BLASTn comparison against GenBank reference sequences. Multiple sequence alignments were generated using MEGA Software 11.0.13. For phylogenetic analysis, the optimal nucleotide substitution model for each dataset was selected based on the lowest Bayesian information criterion score using the model selection function implemented in MEGA 11. Maximum-likelihood phylogenetic trees were then constructed in MEGA 11, and branch support was assessed using 1000 bootstrap replicates. The Kimura 2-parameter model was applied to *Anaplasma* spp., *Ehrlichia* spp., and *Bartonella* spp., the Tamura 3-parameter model to *Borrelia* spp., and the General Time Reversible model to *O. tsutsugamushi*. Outgroups were selected based on previous studies as phylogenetically related but distinct taxa for appropriate tree rooting. Specifically, *Rickettsia rickettsii* was used as the outgroup for *Anaplasma* spp. and *Ehrlichia* spp. [28, 29], *Borrelia duttonii* and *Borrelia anserina* (relapsing fever group) for *Borrelia* spp. [30], *Orientia chuto* for *O. tsutsugamushi* [23, 31, 32], and *Brucella abortus* for *Bartonella* spp. [33].

### Statistical analysis

Regional differences in pathogen prevalence were evaluated using Pearson’s chi-square test. To account for multiple comparisons across 12 regions, the Bonferroni correction was applied, with the significance threshold set at 0.0042 (0.05/12). Co-infection patterns among detected pathogens were assessed using pairwise Phi coefficient analyses of pathogen co-occurrence data. Phi coefficient values range from − 1 to + 1, with the sign indicating the direction of association between pathogen pairs. Statistical analyses were conducted as two-tailed tests using SPSS version 25 (IBM Corp., Armonk, NY, USA). A *p*-value ≤ 0.05 was considered statistically significant.

## Results

### Analysis of the prevalence of viral, bacterial, and parasitic pathogens by TAC assay

A total of 160 wild micromammals were subjected to species identification by sequencing of the mitochondrial *Cytochrome b* gene and revealed the presence of two species: *Apodemus agrarius* (Striped field mouse) (90.6%, *n* = 145) and *Crocidura lasiura* (Ussuri white-toothed shrew) (9.4%, *n* = 15). Spleen tissues were screened for various viral, bacterial, and parasitic pathogens using the TAC assay. Viral pathogens were detected at relatively low frequencies, with JUNV detected at 2.5% (*n* = 4) and LCMV at 0.6% (*n* = 1) in *A. agrarius*, and neither virus detected in *C. lasiura*. In contrast, bacterial pathogens exhibited higher prevalence, including *Bartonella* spp. at 78.8% (*n* = 125 in *A. agrarius* and *n* = 1 in *C. lasiura*), *Ehrlichia* spp. at 47.5% (*n* = 72 in *A. agrarius* and *n* = 4 in *C. lasiura*), *Anaplasma* spp. at 46.9% (*n* = 66 in *A. agrarius* and *n* = 9 in *C. lasiura*), *Borrelia* spp. at 36.3% (*n* = 52 in *A. agrarius* and *n* = 6 in *C. lasiura*), *O. tsutsugamushi* at 25.6% (*n* = 41 in *A. agrarius*), *Rickettsia* spp. at 4.4% (*n* = 5 in *A. agrarius* and *n* = 2 in *C. lasiura*), and *Leptospira* spp. at 2.5% (*n* = 4 in *A. agrarius*). Among protozoan pathogens, *Trypanosoma* spp. showed the highest detection rate at 87.5% (*n* = 128 in *A. agrarius* and *n* = 12 in *C. lasiura*), followed by *Cryptosporidium* spp. at 20.0% (*n* = 30 in *A. agrarius* and *n* = 2 in *C. lasiura*) and *Toxoplasma gondii* at 1.3% (*n* = 2 in *A. agrarius*). Overall pathogen positivity rates detected by TAC are summarized in Table 1.

**Table 1:** Detection of pathogens in wild micromammal species using the TaqMan Array Card assay

**Pathogen**		**Positive ports (%)**		**Total (%)**
		***Apodemus agrarius*** (***n = 145)***	***Crociduralasiura (n* = 15)**	
Virus	Junin virus	4 (2.8)	**–**	4 (2.5)
	Lymphocytic choriomeningitis virus	1 (0.7)	**–**	1 (0.6)
Bacteria	*Bartonella* spp.	125 (86.2)	1 (6.7)	126 (78.8)
	*Ehrlichia* spp.	72 (49.7)	4 (26.7)	76 (47.5)
	*Anaplasma* spp.	66 (45.5)	9 (60.0)	75 (46.9)
	*Borrelia* spp.	52 (35.9)	6 (40.0)	58 (36.3)
	*Orientia tsutsugamushi*	41 (28.3)	**–**	41 (25.6)
	*Rickettsia* spp.	5 (2.1)	2 (13.3)	7 (4.4)
	*Leptospira* spp.	4 (2.8)	**–**	4 (2.5)
Protozoan parasite	*Trypanosoma* spp.	128 (88.3)	12 (80.0)	140 (87.5)
	*Cryptosporidium*	30 (20.7)	2 (13.3)	32 (20.0)
	*Toxoplasma gondii*	2 (1.4)	**–**	2 (1.3)

### Validation of pathogen prevalence by conventional PCR

To obtain genetic sequences from TAC-positive samples, confirmatory conventional PCR was performed. Viral pathogens, including JUNV and LCMV, were not confirmed sequence by conventional PCR. Among bacterial pathogens, partial confirmation was achieved for *Bartonella* spp. at 46.3% (*n* = 74 in *A. agrarius*), *Anaplasma* spp. at 21.3% (*n* = 32 in *A. agrarius* and *n* = 2 in *C. lasiura*), *Ehrlichia* spp. at 13.8% (*n* = 20 in *A. agrarius* and *n* = 2 in *C. lasiura*), *Borrelia* spp. at 10.6% (*n* = 17 in *A. agrarius*), and *O. tsutsugamushi* at 8.1% (*n* = 13 in *A. agrarius*) (Table 2).

**Table 2:** Regional distribution and conventional PCR-based detection rates of five major bacterial pathogens in wild micromammals collected from 12 regions of the Republic of Korea

**Area**	**Region**	**Number tested**	***Bartonella spp.***	***Anaplasma spp.***	***Ehrlichia spp.***	***Borrelia spp.***	***Orientia tsutsugamushi***
Northern	Goseong	18	10 (55.6)	5 (27.8)	6 (33.3)	3 (16.7)	1 (5.6)
	Pocheon	19	6 (34.6)	1 (5.3)	1 (5.3)	2 (10.5)	3 (15.8)
	Donghae	17	9 (52.9)	5 (29.4)	4 (23.5)	5 (29.4)	**–**
	Yeoju	30	19 (63.3)	**–**	**–**	2 (6.7)	**–**
Central	Sejong	12	6 (50.0)	7 (58.3)	1 (8.3)	1 (8.3)	3 (25.0)
	Uiseong	6	3 (50.0)	1 (16.7)	2 (30.0)	**–**	2 (30.0)
	Cheondo	20	7 (35.0)	7 (35.0)	1 (5.0)	1 (5.0)	4 (20.0)
	Jeongeup	14	2 (14.3)	4 (28.6)	2 (14.3)	2 (14.3)	**–**
Southern	Goheung	13	6 (46.2)	4 (30.8)	3 (23.1)	1 (7.7)	**–**
	Haenam	3	**–**	**–**	**–**	**–**	**–**
	Jeju	1	1 (100.0)	**–**	**–**	**–**	**–**
	Seogwipo	7	5 (71.4)	**–**	2 (28.6)	**–**	**–**
Total (%)		160	74 (46.3)	34 (21.3)	22 (13.8)	17 (10.6)	13 (8.1)

All pathogens except *Borrelia* spp. (*p* = 0.343) exhibited statistically significant regional variation (*p* < 0.001) after Bonferroni correction (adjusted α = 0.0042)

**Fig. 1:**
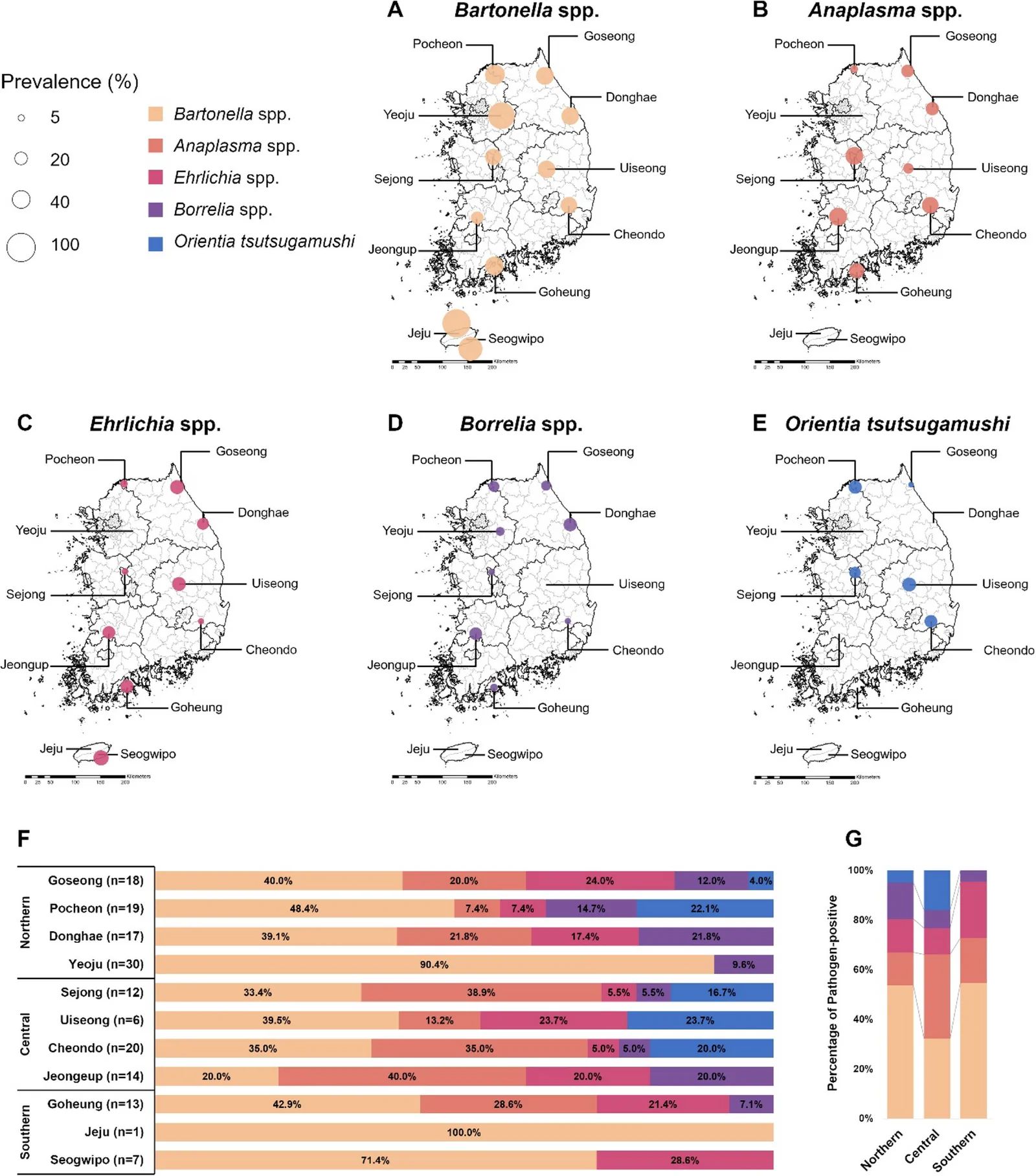
Geographic distribution and regional prevalence of bacterial pathogens detected in wild micromammals by conventional PCR. **A**–**E** Spatial distribution of individual bacterial pathogens, including *Bartonella* spp., *Anaplasma* spp., *Ehrlichia* spp., *Borrelia* spp., and *Orientia tsutsugamushi*. Colored circles represent each pathogen, and the circle area is proportional to pathogen prevalence (%) at each sampling site. Sample sizes (*n*) for each collection site are provided in panel **F** to facilitate interpretation of prevalence estimates. Because some locations had limited sample sizes, prevalence estimates from sites with *n* < 5 should be interpreted with caution. **F** Prevalence of detected pathogens across sampling regions is presented as horizontal stacked bar graphs. **G** Relative composition of pathogens in the northern, central, and southern regions is shown as stacked bar graphs, illustrating differences in pathogen distribution patterns

Regional comparisons revealed that *Bartonella* spp. were most prevalent in Yeoju (63.3%), excluding Jeju Island because of limited sample size, whereas *Anaplasma* spp. showed the highest prevalence in Sejong (58.3%). *Ehrlichia* spp. were most prevalent in Goseong (33.3%), *Borrelia* spp. in Donghae (29.4%), and *O. tsutsugamushi* in Uiseong (30.0%), followed by Sejong (25.0%). All pathogens except *Borrelia* spp. exhibited significant regional variations (*p* < 0.001), which remained significant after Bonferroni correction (adjusted α = 0.0042). *Borrelia* spp. were evenly distributed across regions and showed no significant regional difference (*p* = 0.343) (Table 2). The regional distribution of pathogen detection is shown in Fig. 1.

### Molecular phylogeny and pathogen identification

**Fig. 2:**
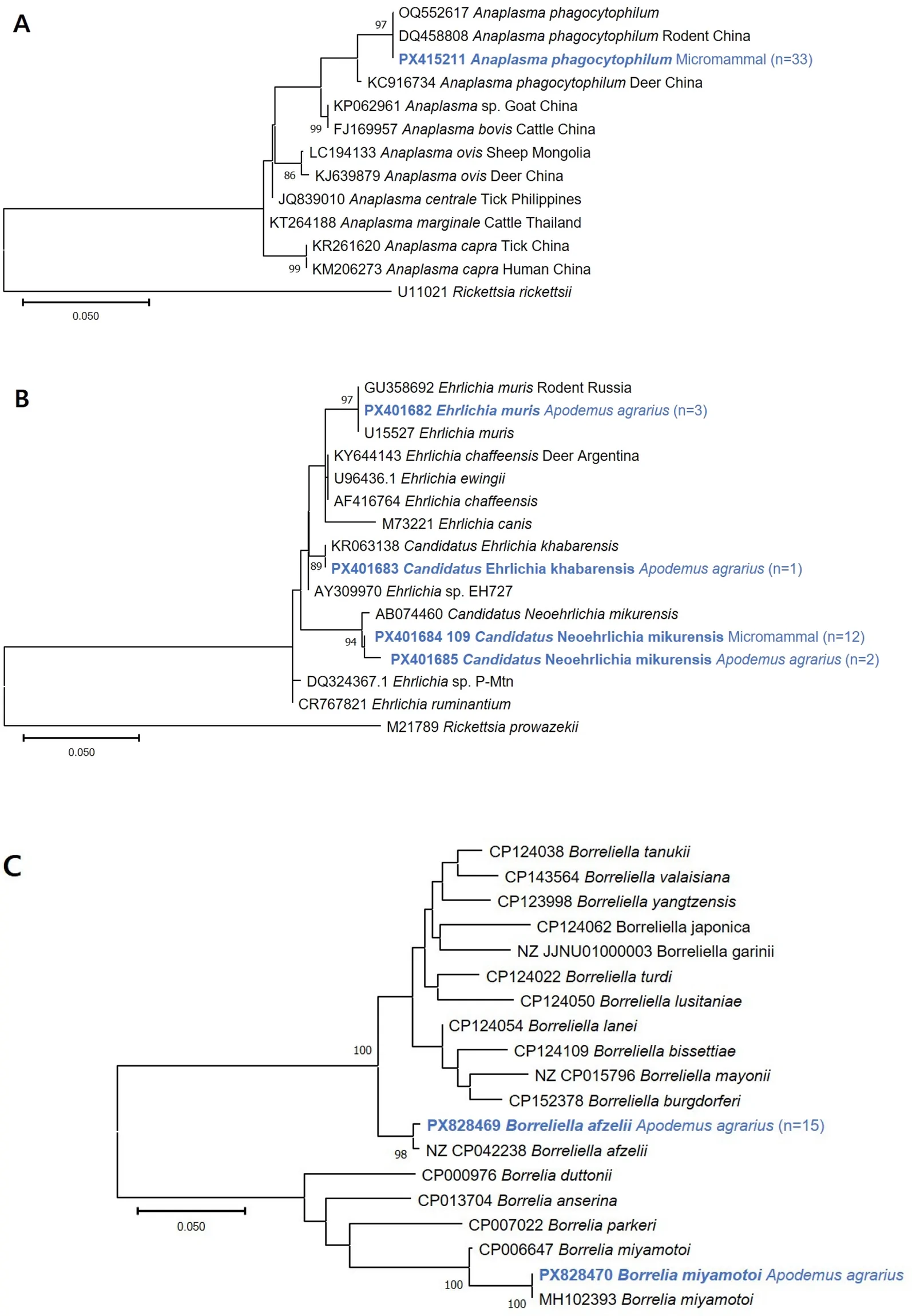

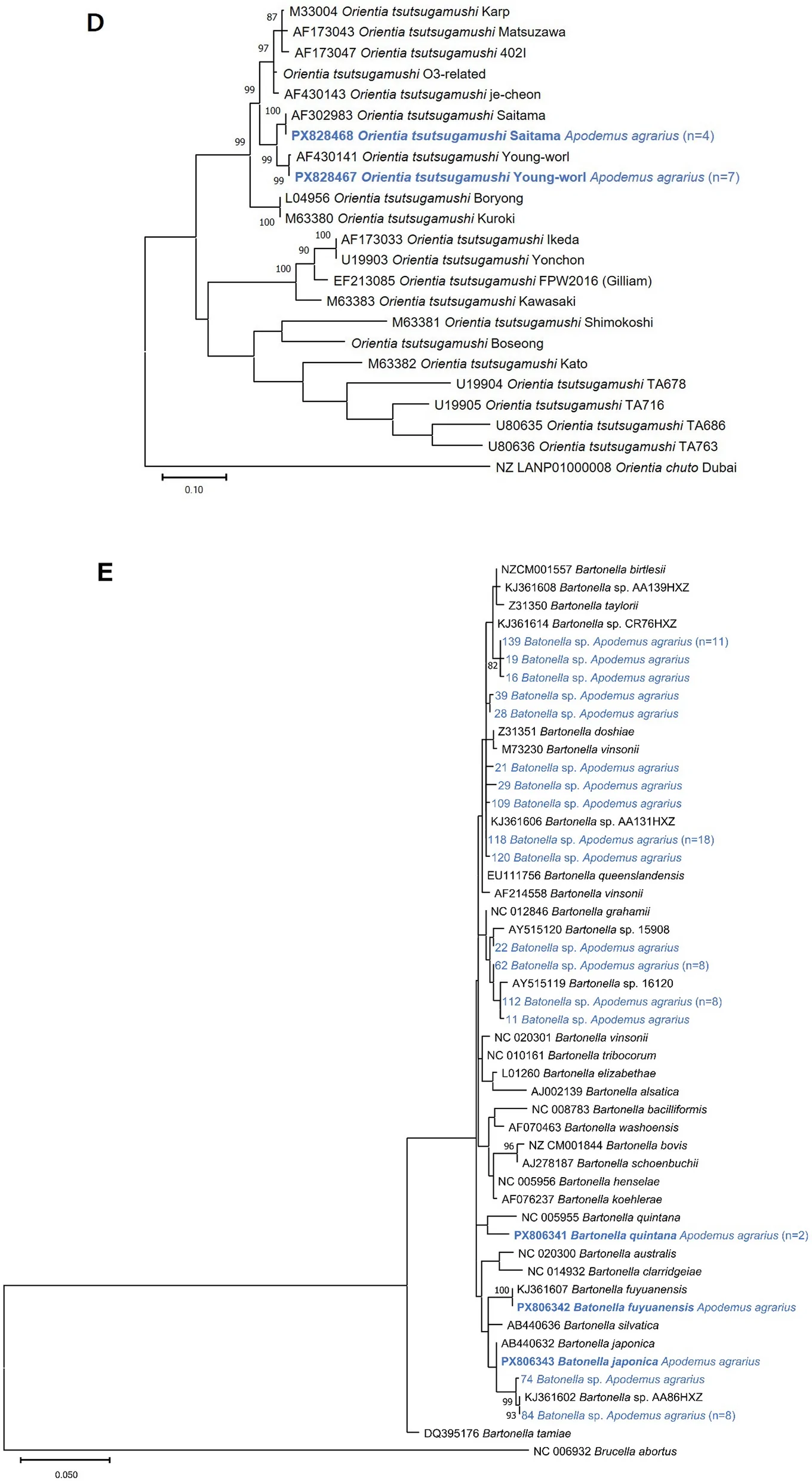
Phylogenetic analysis. Panels **A**–**E** represent phylogenetic trees of *Anaplasma* spp., *Ehrlichia* spp., *Borreliella* and *Borrelia* spp., *Orientia tsutsugamushi*, and *Bartonella* spp. sequences, respectively. Sequences obtained in this study are highlighted in blue with corresponding GenBank accession numbers indicated. The phylogenetic tree was constructed using the maximum likelihood method in MEGA 11. Bootstrap support values based on 1000 replicates are indicated at the nodes. An 80.0% cutoff value was applied to the consensus tree. The number of sequences (n) with corresponding identical sequences is shown when the same sequence was identified in more than one sample

Phylogenetic analysis was performed to confirm the taxonomic identity of the obtained pathogen sequences. All *Anaplasma* 16S rRNA sequences clustered with *A. phagocytophilum* (PX415211, *n* = 33;* n* = 31 in *A. agrarius* and *n* = 2 in *C. lasiura*), showing 100.0% sequence identity (Fig. 2A). *Ehrlichia* 16S rRNA sequences grouped with *E. muris* (PX401682, *n* = 3 in *A. agrarius*), *Candidatus* Ehrlichia khabarensis (PX401683, *n* = 1 in *A. agrarius*), and *Candidatus* Neoehrlichia mikurensis (PX401684, *n* = 12; *n* = 10 in *A. agrarius* and *n* = 2 in *C. lasiura* and PX401685, *n* = 2 in *A. agrarius*), exhibiting 100.0%, 100.0%, and 99.3–100.0% identity to GenBank reference sequences, respectively (Fig. 2B). *Borrelia flaB* sequences formed clades with reference sequences of *Borreliella afzelii* (PX828469, *n* = 15 in *A. agrarius*) (previously named *Borrelia afzelii*) [34] and *Borrelia miyamotoi* (PX828470 in *A. agrarius*), showing 99.4–100.0% and 100.0% identity, respectively (Fig. 2C). *Orientia* 56-kDa *tsa* gene sequences formed Saitama (PX828468, *n* = 4 in *A. agrarius*) and Young-worl (PX828467, *n* = 7 in *A. agrarius*) clades, with 99.8–100.0% sequence similarity to reference strains (Fig. 2D). *Bartonella* sequences based on the 16S rRNA gene formed multiple distinct clades, with representative sequences clustering with *Bartonella quintana* (PX806341, *n* = 2 in *A. agrarius*), *B. fuyuanensis* (PX806342 in *A. agrarius*), and *B. japonica* (PX806343 in *A. agrarius*), showing 98.7–100.0%, 100.0%, and 100.0% identity with the respective reference sequences (Fig. 2E). In contrast, sequences classified as *Bartonella* sp. formed several independent branches that did not cluster with known species, indicating the presence of genetically distinct lineages.

## Discussion

This study represents one of the most comprehensive molecular surveillance efforts to date on micromammal-borne zoonotic pathogens in the ROK. A total of 160 wild micromammals collected from 12 regions were screened for viral, bacterial, and protozoan pathogens using the high-throughput TAC platform. The detection of a broad range of pathogens provides critical baseline data for elucidating the prevalence and diversity of zoonotic agents at a national scale and underscores the importance of multiplex screening tools.

Micromammals exhibit organ-specific tissue tropism, with pathogen detection rates varying according to tissue characteristics [35]. Previous studies have reported that the spleen yields higher detection rates for systemic bacterial and protozoan infections compared with other tissues, supporting its use as the primary specimen for this investigation [27, 36]. However, the spleen is not the primary target organ for all pathogens, particularly many viruses, which may exhibit tissue-specific tropism for the lung, brain, gastrointestinal tract, or blood [37]. Such viral tissue tropism may reduce detection sensitivity in spleen-based surveillance approaches. This study was conducted using spleen samples due to practical constraints in an initial nationwide survey. Nevertheless, future studies should incorporate additional tissues, including lung, brain, intestine, skin, and blood, to better characterize tissue-specific pathogen distributions and to evaluate the generalizability of spleen-based surveillance.

In the TAC results, *Bartonella* spp. exhibited a markedly different prevalence between *A. agrarius* (86.2%) and *C. lasiura* (6.7%). This discrepancy may be attributed to a combination of host-related ecological factors and methodological considerations. *Bartonella* spp. are arthropod-borne bacteria primarily transmitted by fleas and ticks, and their maintenance and transmission within wildlife populations are influenced by host specificity, reservoir competence, and host–vector interactions [38, 39]. A previous study has demonstrated that *Bartonella* lineages frequently exhibit host-associated patterns, suggesting that differences among host species may contribute to variation in infection prevalence [40]. In addition, *A. agrarius* has been reported as an important reservoir host for *Bartonella* spp. in the ROK, with consistently high prevalence rates observed in a previous survey [41]. Therefore, the substantially higher prevalence detected in *A. agrarius* in the present study may reflect differences in host suitability or ecological interactions relative to *C. lasiura*. However, the relatively small sample size of *C. lasiura* (*n* = 15) increases the potential for sampling bias and limits the precision of prevalence estimates. Consequently, the low detection rate observed in this species should be interpreted with caution because it cannot be ruled out that the true prevalence of *Bartonella* infection was underestimated.

In addition, co-infection patterns among detected pathogens were explored using Phi coefficient correlation analyses (Supplementary Figure S1) [42]. The heatmap revealed both positive and negative associations among pathogen pairs, indicating diverse patterns of pathogen co-occurrence within micromammal hosts. These findings provide preliminary insights into pathogen community structure and potential pathogen–pathogen interactions, although the observed associations should be interpreted cautiously because they do not necessarily imply causal relationships. Further studies incorporating larger sample sizes and longitudinal surveillance data are needed to clarify the ecological and epidemiological significance of these associations.

Regional differences in detection rates were observed for *Anaplasma*, *Bartonella*, *Ehrlichia*, and *O. tsutsugamushi* (Fig. 1G and Table 2). *Bartonella* spp. showed relatively higher prevalence in the northern and southern regions, whereas the prevalence of both *Anaplasma* spp. and *O. tsutsugamushi* was slightly higher in the central region. In contrast, *Borrelia* spp. displayed a relatively uniform distribution across regions, indicating widespread nationwide presence irrespective of local ecological variation. Detection of zoonotic pathogens on Jeju Island, including Jeju and Seogwipo, as well as across the mainland further indicates that micromammal-borne disease risks are broadly distributed throughout the country. These findings provide foundational data for future nationwide and region-specific surveillance of zoonotic diseases [8, 10, 30, 43].

Phylogenetic analyses revealed substantial genetic diversity among detected pathogens. All *Anaplasma* sequences were identified as *A. phagocytophilum* (Fig. 2A), supporting its predominance in micromammals in the ROK [10]. *Borreliella afzelii* and *Borrelia miyamotoi*, both transmitted by *Ixodes* ticks commonly parasitizing wild micromammals and recognized as zoonotic pathogens, were also detected (Fig. 2C), confirming their relevance to public health concerns both domestically [8, 30, 44] and globally [45]. For *O. tsutsugamushi*, only the Saitama and Young-worl strains were identified (Fig. 2D), both previously reported in wild micromammals and trombiculid mites in the ROK [32, 46, 47]. The genus *Ehrlichia* exhibited notable diversity, with multiple genospecies detected, including *E. muris*, *C.* E. khabarensis, and *C.* N. mikurensis (Fig. 2B). Domestic studies have shown that *E. muris* [10] and *C.* N. mikurensis [28] are tick-borne pathogens with micromammals serving as their primary reservoirs and that these pathogens have the potential to infect humans. Notably, *C.* E. khabarensis was detected for the first time in the ROK. This species was originally described in wild micromammals in Russia in 2015 [48] and has since been reported in multiple regions of Asia [49]. Several *Bartonella* species were also identified, consistent with previous reports in *A. agrarius* and ticks in the ROK [50]. These included *B. quintana* [51], *B. fuyuanensis*, and *B. japonica* [52] (Fig. 2E), all commonly associated with micromammals, ticks, and fleas. Notably, *B. fuyuanensis* has not been previously reported in the ROK, indicating that its distribution in local micromammal populations warrants further investigation. Collectively, the detection of diverse and potentially novel pathogens indicates a more complex microbial landscape within Korean micromammal populations than previously recognized [10, 28, 53], emphasizing the need for continued surveillance, pathogen isolation, and molecular characterization to better understand epidemiology and zoonotic risk.

This study has several limitations. First, although the TAC platform offers the major advantage of high analytical sensitivity, this also increases the risk of false-positive results. To minimize this concern, conventional PCR was used to validate TAC-positive detections. Amplification signals for JUNV, LCMV, *Rickettsia* spp., and *Leptospira* spp. were observed on the TAC platform; however, these findings could not be confirmed by subsequent conventional PCR and sequencing (Tables 1 and 2). Moreover, less than half of the TAC-positive samples were confirmed by conventional PCR, indicating that TAC results should be interpreted with caution. For example, *Bartonella* spp. was confirmed in 74 of 126 samples, *Ehrlichia* spp. in 22 of 76 samples, *Anaplasma* spp. in 34 of 75 samples, *Borrelia* spp. in 17 of 58 samples, and *O. tsutsugamushi* in 13 of 41 samples (Tables 1 and 2). In principle, statistical approaches such as Bayesian correction can help estimate true prevalence in the absence of a gold standard by accounting for test sensitivity and specificity [54]. However, reliable prior information regarding the sensitivity and specificity of the TAC assays for each of the 45 pathogens analyzed in this study was not available, limiting the practical application of Bayesian correction. Therefore, such analyses were not performed. Consequently, the true prevalence of some pathogens may have been overestimated due to discrepancies between TAC and confirmatory assays. These discrepancies may reflect RNA instability, low viral or bacterial loads, or differences in genomic target regions between the TAC and conventional PCR assays. Accordingly, the findings of this study should be interpreted as results derived from a high-sensitivity TAC-based screening approach.

Furthermore, since specificity issues with the TAC primers and probes were also suspected as a potential source of these discrepancies, a preliminary validation experiment was conducted to evaluate this possibility. A biological matrix-based spike-in model was developed using homogenates of pathogen-free *Aedes albopictus* (striped forest mosquito) maintained under approved laboratory conditions, which served as a non-infected biological matrix [55]. Cultured SFTSV was then spiked into this homogenate to generate a positive spike-in model for validation. In parallel, well-defined positive controls were used to further assess the specificity and analytical accuracy of the TAC platform. The positive controls included synthetic oligonucleotide DNA reference material (CUSTOM CONTROL 20; Thermo Fisher, Waltham, MA, USA), representing 20 of the 45 targeted pathogens on the TAC platform (Supplementary Table S3). In addition, the assay incorporated multiple internal controls to ensure reliability including organism-based, spike-in, and endogenous controls (Supplementary Table S3). These controls collectively confirmed the analytical performance of the TAC platform. The results showed that TAC accurately detected only the intended targets, with no cross-reactivity or false-positive signals observed (data not shown). This confirmed the specificity of the TAC primers and probes and indicated that the TAC platform itself was properly designed. Therefore, while the possibility of false positives due to various factors cannot be entirely excluded, the likelihood of cross-reactivity inherent to the TAC platform appears to be low. Nevertheless, given that LCMV has been reported in China and Japan [56–59], and that *Rickettsia* spp. [10] and *Leptospira* spp. [8] have been documented in wild micromammals in the ROK, these observations underscore the need for continued surveillance and molecular investigation of these pathogens domestically.

In addition to the risk of false-positive results, the possibility of false-negative findings should also be considered. When pathogen loads fall below the detection limit, target pathogens may not be detected, potentially leading to an underestimation of the true prevalence. Accordingly, among the 45 pathogens included in the TAC panel, a preliminary assessment of analytical sensitivity was performed for SFTSV and *O. tsutsugamushi*, which were the targets in this field investigation. This assessment was not intended to determine absolute limits of detection through serial dilution; rather, it was conducted by applying a limited number of additional dilution steps based on the detection range of the conventional assays. Under these conditions, SFTSV was detectable at approximately 100-fold lower concentrations compared to conventional qPCR, while *O. tsutsugamushi* showed a tendency to be detected at approximately twofold lower concentrations compared to conventional nested PCR. These findings suggest that the TAC platform may offer improved analytical sensitivity compared to conventional methods. Although false-negative results cannot be completely excluded due to the methodological limitations of this evaluation, the results indicate that the TAC platform is capable of detecting low-level pathogen loads with enhanced sensitivity.

Second, protozoan pathogens detected by the TAC assay were not subjected to confirmatory conventional PCR because the quantity and quality of RNA extracted from some spleen samples were insufficient for reliable amplification. In addition, as this study primarily focused on viral and bacterial pathogens of zoonotic importance, protozoan detections were considered beyond the primary validation scope. Notably, the detection of *Cryptosporidium* in spleen samples is biologically unexpected, as this protozoan primarily infects intestinal epithelial cells and is not known to invade lymphoid organs. Therefore, these observations are more likely to reflect sample contamination or carryover from intestinal contents rather than true systemic infection [60]. However, because intestinal contents or fecal samples were not analyzed in parallel in the present study, it was not possible to directly determine whether the detected *Cryptosporidium* DNA originated from true intestinal infection or from contamination during sample collection and processing. This limitation should be considered when interpreting the protozoan detection results. In addition, rare reports have documented the molecular detection of *Cryptosporidium* DNA in the blood and cerebrospinal fluid of immunocompromised patients [61]. In the present study, it is also possible that blood contacted the spleen during tissue collection, allowing *Cryptosporidium* DNA present in the blood to be carried over into the spleen samples, which cannot be entirely excluded. Nevertheless, the TAC assay provides high sensitivity and specificity for multiplex pathogen detection, and these protozoan detection results can be considered preliminary. Future studies should perform confirmatory analyses and molecular characterization of protozoan pathogens, with careful consideration of sample type, to complement the present findings and improve our understanding of micromammal-borne zoonotic agents in the ROK.

Third, sequences classified as *Bartonella* sp. clustered into branches with relatively low bootstrap support, reflecting limited phylogenetic resolution based solely on 16S rRNA gene analysis (Fig. 2E). The use of a single genetic marker may restrict the accuracy of genotypic classification and phylogenetic inference. Although the application of additional genetic markers, such as *gltA*, *ftsZ*, and *rpoB* [62], would enable higher-resolution analysis, these investigations were not performed in the present study. Therefore, to more clearly elucidate the genetic diversity and taxonomic status of the *Bartonella* lineages identified in this study, further PCR amplification and sequencing targeting these loci, as well as concatenated phylogenetic analyses integrating multiple gene sequences, will be necessary in future studies [63, 64].

Fourth, although orthohantaviruses (*Orthohantavirus hantanense* and *O. jejuense*) are well-documented micromammal-borne pathogens in the ROK [65], they were not detected by the TAC platform in this study. This absence should be interpreted with caution and does not necessarily indicate true absence in the sampled populations. Possible explanations include limited sample size, low viral loads, and tissue selection. Especially, spleen tissues were primarily analyzed in this study, whereas orthohantaviruses are more readily detected in lung or kidney tissues [66]. In addition, the difference between targeted design of the TAC assay and genetic diversity among circulating orthohantavirus strains may have reduced primer and probe matching efficiency. These considerations highlight the need for expanded sampling strategies, inclusion of appropriate target tissues, and complementary molecular diagnostic approaches to accurately assess orthohantavirus circulation in micromammal populations.

## Conclusions

This study provides essential baseline data on the distribution and diversity of micromammal-borne zoonotic pathogens in the ROK. These findings underscore the importance of the TAC platform in enhancing our understanding of the prevalence and diversity of zoonotic diseases at the national scale. Given the growing risk of zoonotic spillover events driven by factors such as urban expansion into natural habitats, climate change, and increased human–animal interactions [67, 68], strengthening wildlife disease surveillance is imperative for early warning and effective prevention. The application of TAC in this study supports timely risk assessment and public health preparedness. Importantly, the interpretation of these findings should consider the local ecology of vectors, such as trombiculid mites and ticks parasitizing micromammals, as well as potential human exposure in the same regions. Integrating pathogen prevalence data from micromammals with vector abundance and infection rates in local human populations enables a multidimensional understanding of potential transmission pathways and epidemiological trends. Such an approach enhances the capacity for integrated surveillance and strengthens early warning systems for emerging zoonotic threats, allowing for targeted interventions and resource allocation. Future studies should integrate ecological and seasonal variables, expand vector surveillance, and employ advanced approaches such as metagenomic or whole-genome sequencing to identify novel or emerging pathogens. In addition, isolation of viable pathogens will be essential for clarifying pathogenic potential and relevance to human health. Ultimately, these efforts will require coordinated collaboration across human, animal, and environmental health sectors, consistent with the One Health framework, to inform surveillance strategies, guide policy-making, and strengthen national capacity to respond to emerging infectious disease threats.

## Supplementary Information

Supplementary Material 1

Supplementary Material 2

## Data Availability

Data supporting the conclusions of this article are included within the article.
