# Supplementary material for: Broad-spectrum molecular detection of zoonotic agents in wild micromammals: a nationwide surveillance study in the Republic of Korea
Source: Parasit Vectors. 2026 Jul 26;19:435. doi: 10.1186/s13071-026-07585-w (PMC13647823; doi:10.1186/s13071-026-07585-w)
Supplement: Supplementary file 1 [file 13071_2026_7585_MOESM1_ESM.docx]

**Supplementary Figure S1.** Heatmap showing pairwise pathogen associations based on Phi coefficient analysis in *Apodemus agrarius* (A) and *Crocidura lasiura* (B). Cell values represent Phi coefficient estimates. Positive values indicate positive associations between pathogen pairs (co-occurring more frequently than expected), whereas negative values indicate negative associations (co-occurring less frequently than expected). Phi coefficient values range from −1 to +1, with larger absolute values indicating stronger associations and values closer to 0 indicating weaker associations. Heatmap colors correspond to Phi coefficient values, with red indicating positive associations and blue indicating negative associations. Because the sample size of C. lasiura was limited (*n* = 15), associations observed in this host species should be interpreted with caution.
