# Supplementary material for: Broad-spectrum molecular detection of zoonotic agents in wild micromammals: a nationwide surveillance study in the Republic of Korea
Source: Parasit Vectors. 2026 Jul 26;19:435. doi: 10.1186/s13071-026-07585-w (PMC13647823; doi:10.1186/s13071-026-07585-w)
Supplement: Supplementary file 2 [file 13071_2026_7585_MOESM2_ESM.docx]

**Supplementary Table S1.** Pathogen panel included in the TAC assay for simultaneous detection. The table lists pathogens assigned to each port in the left and right columns.

| Port | |  | Port | |
| --- | --- | --- | --- | --- |
| Left | |  | Right | |
| 24 | *Rickettsia prowazekii* |  | GAPDH | 48 |
| 23 | *Rickettsia typhi* |  | *B. atrophaeus,* Xeno | 47 |
| 22 | *Rickettsia* spp. |  | *Entamoeba histolytica* | 46 |
| 21 | *Borrelia* spp. |  | *Angiostrongylus cantonensis* | 45 |
| 20 | *Rickettsia tsutsugamushi* AKA *Orientia tsutsugamushi* |  | *Capillaria hepatica* | 44 |
| 19 | *Ehrlichia* spp. |  | *Brachylaima* spp. | 43 |
| 18 | *Anaplasma* spp. |  | *Fasciola* spp. | 42 |
| 17 | Cowpox virus |  | *Schistosoma* spp. | 41 |
| 16 | Colorado tick fever virus |  | *Echinococcus* spp. | 40 |
| 15 | Junin virus AKA Argentinian mammarenavirus |  | *Hydatigera taeniaeformis* | 39 |
| 14 | Powassan virus |  | *Giardia* spp. | 38 |
| 13 | Borna disease virus |  | *Cryptosporidium* spp. | 37 |
| 12 | Songling virus |  | *Trichinella* spp. | 36 |
| 11 | 18s rRNA |  | *Toxoplasma gondii* | 35 |
| 10 | Tacheng tick virus |  | *Leishmania* spp. | 34 |
| 9 | Kyasanur Forest disease virus |  | *Trypanosoma* spp. | 33 |
| 8 | Lassa virus AKA Lassa mammarenavirus |  | *Theileria* spp. | 32 |
| 7 | Omsk hemorrhagic fever virus |  | *Babesia* spp. | 31 |
| 6 | Hepatitis E virus |  | *Yersinia pestis* | 30 |
| 5 | Lymphocytic choriomeningitis mammarenavirus |  | *Streptococcus moniliformis* | 29 |
| 4 | Crimean-Congo hemorrhagic fever virus |  | *Leptospira* spp. | 28 |
| 3 | Tick-borne encephalitis virus |  | *Francisella tularensis* | 27 |
| 2 | Hantavirus |  | *Coxiella burnetii* | 26 |
| 1 | Severe fever with thrombocytopenia syndrome virus (SFTSV) |  | *Bartonella* spp. | 25 |

**Supplementary Table S2.** Primer sets used for conventional PCR confirmation of viral and bacterial pathogens.

| Pathogen | Target gene | Primer name | Sequence (5' → 3') | Size  (bp) | Ref. |
| --- | --- | --- | --- | --- | --- |
| Junin virus | S segment | JUNV 247F | TCACAGCGTCACTCTTCCTTC | 346 | Thermo Fisher Scientific |
|  |  | JUNV 620R | AGTCAGTGCTAGATGGGAAGC |  |  |
|  |  | JUNV 418F | GGCGTAGAGGACACTAAGACC | 371 |  |
|  |  | JUNV 789R | ACATCTGAACAAGCCAGGCA |  |  |
|  | L gene | LVL-3359D/G | AGAATYAGTGAAAGGGARAGYAATTC | 396 | [69] |
|  |  | LVL-3754A/D | CACATCATTGGTCCCCATTTACTRTGATC |  |  |
| Lymphocytic choriomeningitis virus | S segment | 1817V-LCM | AIATGATGCAGTCCATGAGTGCACA | 660 | [70] |
|  |  | 2477C-LCM | TCAGGTGAAGGRTGGCCATACAT |  |  |
|  |  | 1902V-LCM | CCAGCCATATTTGTCCCACACTTT | 445 |  |
|  |  | 2346C-LCM | AGCAGCAGGYCCRCCTCAGGT |  |  |
|  | S segment | LCMV-1638F | TGGAGAGTCAGGGAGGCC | 650 | [71] |
|  |  | LCMV-1010C | TCNGGNGARGGNTGGCC |  |  |
|  |  | LCMV-1696 | ATNATGCARTCCATRAGWGCACA |  |  |
|  | GP  (S segment) | LCMV-001 | CGCACCGGGGATCCTAGGCTT | 907 | [72] |
|  |  | LCMV-R1 | TCGTAGCATGTCACAG |  |  |
|  |  | LCMV-DM1 | GAATTCTATCCAGTAAAAGGATGG |  |  |
|  |  | LCMV-RC1 | GAGCTCTGCAGCAAGGATCATCC |  |  |
|  | NP  (S segment) | LCMV-2818 | GATGAATTCTTCACATCCAAACCC | 207 |  |
|  |  | LCMV-3060 | GATCCGAATTCTACATCAAAG |  |  |
|  |  | LCMV-NP5'Ⅰ | TACTACACCACTTGCACCCTG |  |  |
|  |  | LCMV-NP3'Ⅰ | TCTGAAAGTGGGAAGACTTAG |  |  |
| *Bartonella* spp. | 16s rRNA | A-proteo | AGAGTTTGATMCTGGCTCAGA | 1210 | [73] |
|  |  | r-Alpha-sh | GTAGCACGTGTGTAGCCCA |  |  |
|  |  | Bart | CACTCTTTTAGAGTGAGCGGCAA | 990 |  |
|  |  | r-BH | CCCCCTAGAGTGCCCAACCA |  |  |
| *Ehrlichia* spp. | 16s rRNA | AE1-F | AAGCTTAACACATGCAAGTCGAA | 1406 | [74] |
|  |  | AE1-R | AGTCACTGACCCAACCTTAAATG |  |  |
|  |  | HE1 | CAATTGCTTATAACCTTTTGGTTATAAAT | 390 |  |
|  |  | HE3 | TATAGGTACCGTCATTATCTTCCCTAT |  |  |
| *Anaplasma* spp. | 16s rRNA | AE1-F | AAGCTTAACACATGCAAGTCGAA | 1406 | [75] |
|  |  | AE1-R | AGTCACTGACCCAACCTTAAATG |  |  |
|  |  | EE3 | GTCGAACGGATTATTCTTTATAGCTTGC | 926 | [75, 76] |
|  |  | EE4 | CCCTTCCGTTAAGAAGGATCTAATCTCC |  |  |
| *Borrelia* spp. | *flaB* | BflaPAD | GATCARGCWCAAYATAACCAWATGCA | 451 | [77, 78] |
|  |  | BflaPDU | AGATTCAAGTCTGTTTTGGAAAGC |  |  |
|  |  | BflaPBU | GCTGAAGAGCTTGGAATGCAACC | 347 |  |
|  |  | BflaPCR | TGATCAGTTATCATTCTAATAGCA |  |  |
| *Orientia tsutsugamushi* | 56-kDa *tsa* | First F | GCAATATTGCTAGTGCAATGTCTGC | 475 | [45] |
|  |  | First R | ATGCATGCATGRCGCTKCAATTTA |  |  |
|  |  | Second F | ATAGGCCTATAAGTATWGCKGATCG |  |  |
|  |  | Second R | CATCTAGAYGCACTATTAGGCAAA |  |  |
| *Rickettsia* spp. | *gltA* | CS-78 | GCA AGT ATC GGT GAG GAT GTA AT | 381  -401 | [79] |
|  |  | CS-323 | GCT TCC TTA AAA TTC AAT AAA TCA GGA T |  |  |
| *Leptospira* spp. | *rpoB* | *rpoB-1889F* | GTTCCAACATGCAACGYCAR | 1649 | [80] |
|  |  | *rpoB-3537R* | GTTGAAGGATTCRGGRATAC |  |  |
|  |  | *rpoB-2438F* | TYATGCCKTGGGAAGGWTAC | 1023 |  |
|  |  | *rpoB-3460R* | GCATRTCRTCKGACTTGATG |  |  |
|  | *lipL32* | hap1-435F | GGGAATACGTAGAMGTTCG | 1435 | [81] |
|  |  | hap1-1870R | GTTTATAGTAGGTTGAAGCTTG |  |  |
|  |  | L-hap1-217F | CCGTGATTTTCCTAACTAAGG | 848 | [81] |
|  |  | L-hap1-218R | CAGATTACTTAGTCGCGTCAGA |  |  |
|  | *gyrB* | LeptoB2F | TGAGCCAAGAAGAAACAAGCTACA | 500 | [82] |
|  |  | LeptoB504R | MATGGTTCCRCTTTCCGAAGA |  |  |

**Supplementary Table S3.** Positive and internal controls included in the TAC assay for validation.

| Synthetic oligonucleotide DNA reference material (Organism name) | Multiple internal controls (ICs) |
| --- | --- |
| SFTSV, Hepatitis E virus, Tacheng tick virus, Songling virus, Borna disease virus, *Anaplasma* spp., *Ehrlichia* spp., *Bartonella* spp., *Streptococcus moniliformis*, *Babesia* spp., *Theileria* spp., *Trypanosoma* spp., *Trichinella* spp., *Giardia* spp., *Hydatigera taeniaeformis*, *Echinococcus* spp., *Schistosoma* spp., *Fasciola* spp., *Brachylaima* spp., *Capillaria hepatica* | *Bacillus atrophaeus* as an organism-based spike-in IC, Xeno™ synthetic RNA as a spike-in IC, GAPDH as a rat housekeeping gene IC, 18S rRNA as a manufacturer-provided endogenous IC |
